# Hydrogen Oxidation on Stepped Rh Surfaces: µm-Scale versus Nanoscale

**DOI:** 10.1007/s10562-016-1824-4

**Published:** 2016-08-23

**Authors:** M. Datler, I. Bespalov, S. Buhr, J. Zeininger, M. Stöger-Pollach, J. Bernardi, G. Rupprechter, Y. Suchorski

**Affiliations:** 1 Institute of Materials Chemistry, Technische Universität Wien, 1060 Vienna, Austria; 2 University Service Center for Transmission Electron Microscopy, Technische Universität Wien, 1060 Vienna, Austria

**Keywords:** Hydrogen oxidation, Photoemission electron microscopy, Field emission microscopy, Rhodium

## Abstract

**Abstract:**

The catalytic H_2_ oxidation reaction on stepped Rh surfaces in the 10^−6^ mbar pressure range was studied in situ on individual high-Miller-index domains of a polycrystalline Rh foil by PEEM (photoemission electron microscopy) and on a Rh nanotip by FIM/FEM (field-ion/field-emission microscopy). The activity, particularly the tolerance to poisoning by oxygen, was found to strongly depend on the density of steps and defects, as well as on the size of the catalytically active surfaces.

**Graphical Abstract:**

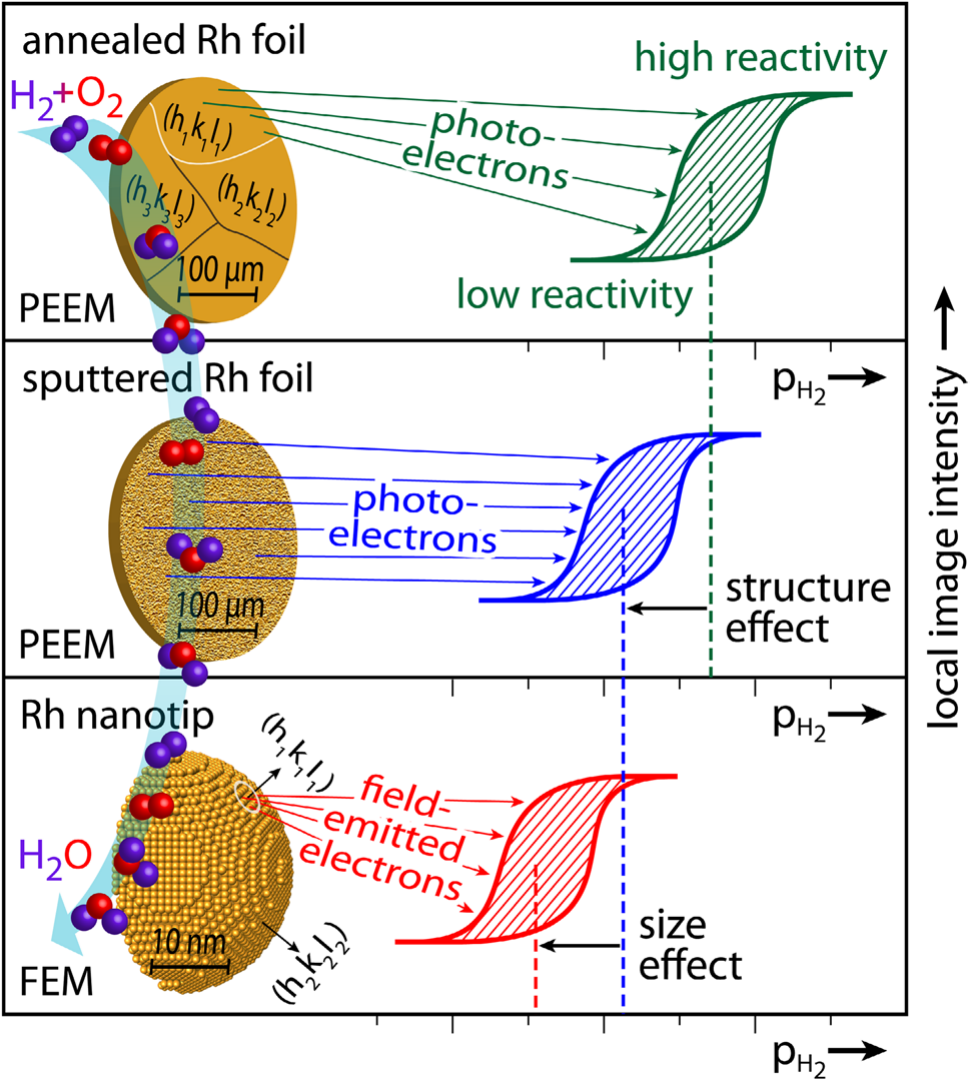

## Introduction

It is well known that the catalytic activity of stepped high-index planes of platinum group metals is generally higher than that of the corresponding low-index planes [1]. This stimulated attempts to synthesize metal nanoparticles terminated by high-index facets [2] as well as studies of catalytic model systems exhibiting stepped surfaces ([3] and references therein). Such studies are usually performed either on single crystal surfaces with high Miller indexes or on curved crystals, for which the step density varies across the sample surface [4–6]. To examine the effect of steps, the first approach requires separate experiments using differently oriented single crystal surfaces. Keeping experimental parameters, such as pressure and temperature, identical in different experiments is, however, sometimes difficult. Applying the second approach, i.e. using curved surfaces, typically yields area-averaged catalytic data (e.g. by mass-spectrometry), unless one is able to resolve the local reaction kinetics on different regions of the curved crystal.

In the present contribution we employ the *novel kinetics by imaging* approach, based on the analysis of PEEM video-files taken during the ongoing reaction to extract locally-resolved kinetic information [7]. The approach is applied to stepped Rh surfaces present on differently oriented grains of a polycrystalline Rh foil. The main advantage of this approach lies in the presence of several µm-sized grains with different step density within the PEEM field of view. This allows a direct comparison of such differently structured surfaces within a single experiment, i.e. under identical experimental conditions. A series of studies of CO oxidation on individual µm-sized low Miller index Pt- and Pd-domains, as well as on supported Pd powder demonstrated the applicability and reliability of this approach [8–10].

For a comparative nm-scale study, the apex of a Rh nanotip, which exhibits a very high curvature, was used, enabling thus to address large differences in the step density. Such a nm-sized apex may also serve as a model of an individual catalytic nanoparticle with a highly stepped surface, as e.g. applied in the recent study by Zeng et al. [11]. In contrary to such a particle, the surface of a nanotip can be prepared in a controlled way by field evaporation and can be characterised by FIM with atomic resolution. Switching to the FEM imaging mode (just by reversing the tip-polarity) the reaction can be imaged in situ on a nanoscale [12]. Applying the same approach as for the µm-sized samples, i.e. analysing the video-files obtained in situ, the local reaction kinetics can be evaluated for individual, differently oriented nanofacets of the tip-apex. As in the case of the PEEM studies mentioned above, such an approach was successfully applied for studying the local kinetics in the catalytic CO oxidation [13]. These studies, performed on a Pt nanotip, allowed to reveal nanosize-caused peculiarities of the reaction, e.g. synchronized kinetic transitions on differently oriented facets of a Pt nanotip.

In the present contribution, we apply a similar comparative approach to H_2_ oxidation on Rh, i.e. we study the reaction kinetics on individual differently oriented domains of a polycrystalline Rh foil by PEEM and compare the obtained results with data obtained for a Rh nanotip by FEM. Although H_2_ oxidation has been studied on Rh single crystals [14–16], the detailed role of the surface structure (including the effect of steps and kinks) and of the domain- and facet-size, as well as of the spatial coupling of the reaction on heterogeneous surfaces, is still not fully understood yet.

## Experimental

The current experiments were performed in two different all-metal UHV setups: (a) a PEEM/XPS setup consisting of separate PEEM and XPS chambers connected with each other by a sample transfer line and (b) a FIM/FEM setup that can be operated either in the FIM mode using Ne as the imaging gas or in the FEM mode (for details see [8] and [12], respectively).

The PEEM/XPS setup is equipped with a PEEM (Staib Instruments), a deuterium discharge UV lamp (photon energy ~ 6.5 eV) for electron excitation, an MS (MKS Instruments), an XPS-system (Phoibos-100 hemispherical energy analyzer and XR 50 twin anode X-ray source, both from SPECS), a high purity gas supply system (O_2_: 99.99 %, H_2_: 99.97 %) and sample preparation facilities for cleaning the sample by argon ion sputtering and subsequent annealing.

**Fig. 1:**
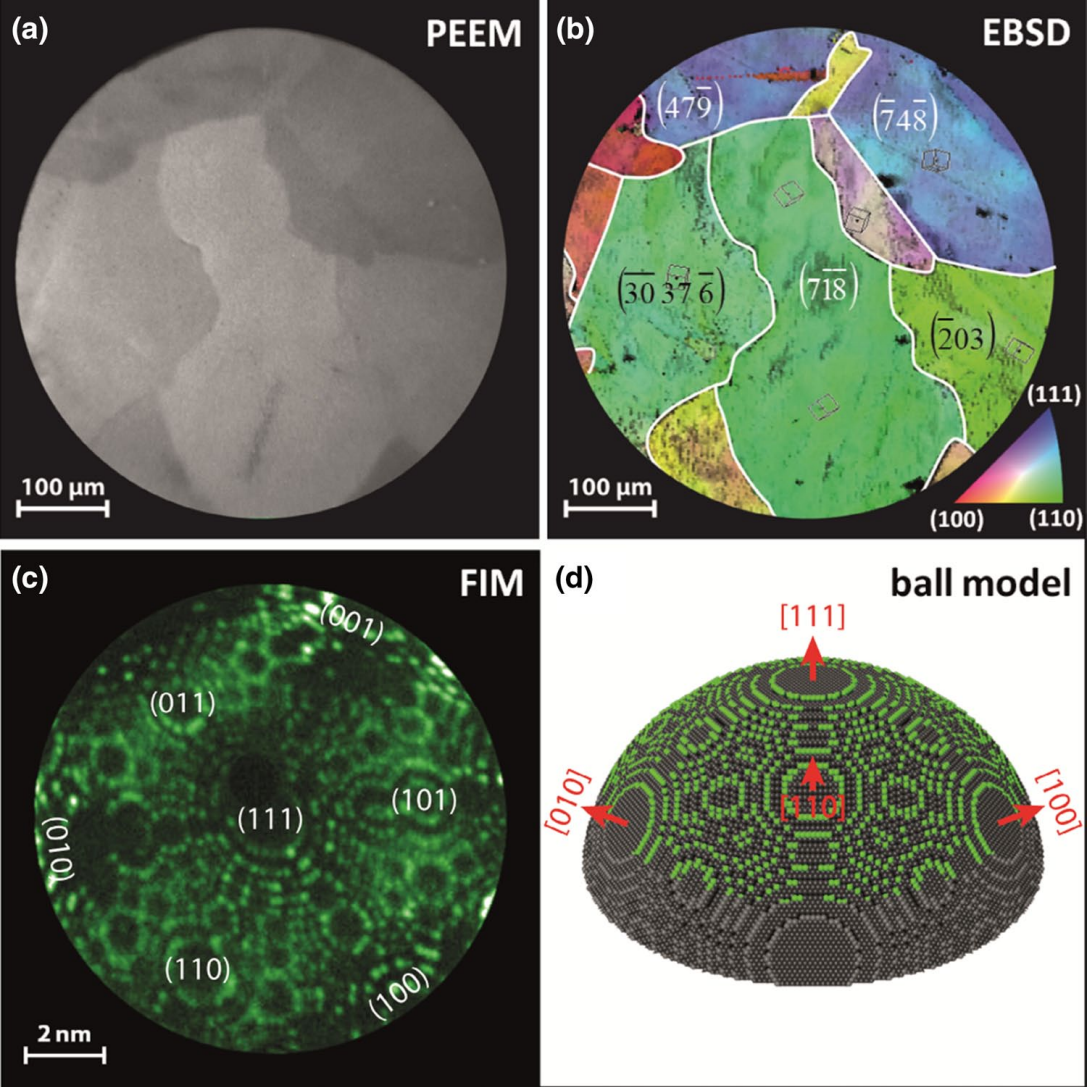
Model systems of stepped Rh surfaces **a** PEEM image of a clean polycrystalline Rh foil surface consisting of μm-sized differently oriented high Miller index domains; **b** EBSD colorcoded map of the same region with indicated crystallographic orientations of individual domains. The inverse pole figure is shown for reference in the *bottom right* corner; **c** Ne + FIM image of the [111]-oriented Rh nanotip; **d** the 3D ball model based on the atomically resolved FIM images. Atoms visible in the FIM micrograph are marked in *green*

The PEEM chamber was used as a flow reactor for H_2_ oxidation on a polycrystalline Rh foil, the reaction was visualized in situ by PEEM and the images were recorded by a CCD camera (Hamamatsu). The PEEM magnification, which allows monitoring the ongoing reaction on individual µm-sized domains of the polycrystalline sample (Fig. 1a) was calibrated by comparison of PEEM images with optical micrographs of the same Rh foil. The sample consisted of a 10 × 12 mm^2^ polished polycrystalline Rh foil of 0.2 mm thickness (Mateck 99.99 %) which was cleaned in UHV by repeated cycles of sputtering with Ar^+^ ions at 1 keV at 300 K and consecutive annealing to 973–1073 K for 30 min. The cleanness of the sample was confirmed by XPS before each single reaction measurement. The sample temperature was measured by a Ni/NiCr thermocouple spot-welded to the sample.

To study the processes on individual µm-sized domains of a polycrystalline foil, their exact crystallographic orientation should be defined first. In the present study this was done by EBSD (electron back scattering diffraction). The EBSD measurements were performed by a field emission scanning electron microscope (FEI Quanta 200F) using standard EBSD conditions and evaluation procedures [17]. The corresponding result is shown in the Fig. 1b, where the same field of view as in the Fig. 1a is shown, but with marked crystallographic orientations, together with the inverse pole figure.

Similar as in the PEEM study, the FIM chamber was used as a flow reactor for H_2_ oxidation on a Rh nanotip. The corresponding UHV system contains a tip assembly, which allows operation in a controlled temperature range of 78–900 K, a gas-supply and a channel-plate/screen assembly used for imaging the ions of either noble (Ne) or reactive (H_2_, O_2_) gases. The Rh nanotip was fabricated by electrochemical etching followed by field evaporation at 77 K in UHV for fine shaping and entire cleaning of the surface under Ne^+^ FIM imaging control. The temperature of the tip was measured by a Ni/NiCr thermocouple spot-welded to its shank. The FIM images during the tip preparation and the FEM images during the ongoing H_2_ oxidation were recorded with the same camera as in the PEEM experiments. In Fig. 1c, the FIM image of the Rh nanotip, obtained with Ne^+^ ions at 77 K and at an applied field of 35 V/nm, is shown. Figure 1d presents a realistic 3D model of the tip apex constructed on the basis of the FIM images. Rhodium atoms, visible in the FIM micrograph, are marked green in the 3D model (only protruding atoms are imaged by FIM, due to the local field based FIM imaging mechanism [18, 19]).

## Results and Discussion

### PEEM Studies

As already mentioned, hydrogen oxidation has been intensively studied on low Miller index single crystal surfaces of Rh [14–16]. These studies revealed the bistable character of the reaction, i.e. an existence of two coexisting steady states (of high and low catalytic activity) with kinetic transitions between them. The transitions are typically manifested by propagating reaction fronts, as, e.g., commonly observed during CO oxidation on single crystals [20]. Such propagating fronts were also observed in the H_2_ oxidation on Rh [14–16, 21]. However, under certain conditions the reaction exhibits a number of additional and unusual features, such as appearance of triangular-shaped reaction fronts on Rh(111) which have been attributed to the formation of subsurface oxygen species [22]. Since our intention was rather to compare the reactive behaviour of µm- and nm-sized stepped Rh surfaces, conditions were chosen, at which the formation of subsurface oxygen is not observed, i.e. in the oxygen pressure range of 10^−7^–10^−6^ mbar and in the temperature range 434–500 K [23].

The bistability in H_2_ oxidation essentially originates from the Langmuir–Hinshelwood mechanism, considering the inequivalence of the adsorption of reactants (hydrogen and oxygen). The bistability shows up as a hysteresis-like behaviour of the reaction rate upon cyclewise variation of control parameters, e.g. the H_2_ pressure (at constant p_O2_ and T). In the hysteresis loop, first a sudden increase of H_2_O production occurs at increasing H_2_ pressure and then a return to the low reaction rate, when one lowers the H_2_ pressure again [15]. The width of the hysteresis loop depends to some extent on the scan rate of the H_2_ pressure (dp_H2_/dt), similarly as it was observed earlier for the CO oxidation [24], therefore the scan rate was reduced till the stable (finite) hysteresis width could be established (usually at <1 × 10^−8^ mbar/s).

**Fig. 2:**
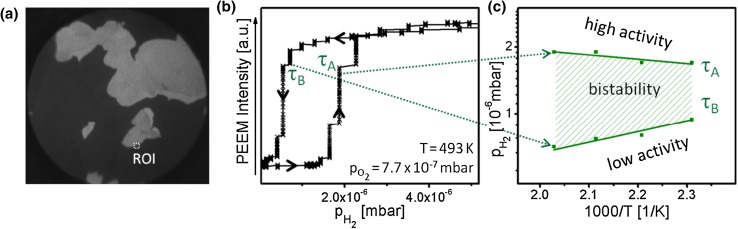
Hydrogen oxidation on µm-sized domains of a polycrystalline Rh foil: **a** PEEM image of the same field of view as shown in Fig. 1a, but taken during the propagation of the reaction front accompanying the kinetic transition τ_A_ in **b** from the low catalytic activity state (*“da*rk” surface) to the high activity state (“*bright*” surface); **b** hysteresis-like curve of the local PEEM intensity recorded during the cyclic variation of the hydrogen pressure at constant temperature of 493 K and an oxygen pressure of 7.7 × 10^−7^ mbar, τ_A_ and τ_B_ mark the kinetic transitions between the steady states of low and high catalytic activity. The ROI for which the PEEM intensity was registered, is marked in (**a**); **c** τ_A_ and τ_B_ values for different temperatures in the range of 433–493 K and constant oxygen pressure of 7.7 × 10^−7^ mbar, plotted in the *p*_H2_ *vs* 1/T coordinates (kinetic phase diagram). Regions of high and low catalytic activity and of bistability are marked

During the PEEM monitoring of the reaction, the PEEM image intensity is governed by the work function of the imaged surface, i.e. by the coverage of reactants. Since also the H_2_O production rate depends in a straight-forward way on the surface coverage of reactants, low image brightness (high work function, oxygen covered surface) corresponds to the catalytically inactive state. In turn, the bright areas are regions of high catalytic activity, i.e. image brighness reflects the catalytic activity. For the image intensity averaged over the whole field of view, this was quantitatively proven by Schaak and Imbihl [15], but since PEEM reflects the spatial distribution of the local work function across the sample, also the spatial distribution of the reaction rate is contained in the PEEM images [7]. In the present case this means that the variations of the *local* PEEM intensity reflect the variations of the *local* H_2_O production rate. Figure 2a, b shows such local PEEM intensity variation for the indicated Rh$(7\bar{1}\bar{8})$ domain, measured during the cyclewise variation of the H_2_ pressure at constant temperature of 493 K and constant oxygen pressure of 7.7 × 10^−7^ mbar, together with a snap-shot of the reaction front propagation. The snap-shot (PEEM frame) in Fig. 2a corresponds to the transition τ_A_ from the low (dark contrast) to the high (bright contrast) catalytic activity. Figure 2c shows the corresponding *kinetic phase diagram* constructed as a plot of transition points τ_A_ and τ_B_ measured for different temperatures in the range of 433–493 K.

This type of illustrating the reaction behaviour allows the identification of the regions of high and low activity, or, respectively, of bistability, at “first glance” and is often used for the CO oxidation reaction [8, 25, 26]. The term “kinetic phase diagram” is justified by the analogy to equilibrium thermodynamics (cooperative phenomena play the crucial role in both equilibrium and nonequilibrium phase transitions [27, 28]); see also the discussion in [25, 26]. Similarly, as for CO oxidation, the diagram in Fig. 2c differentiates the monostable regions of high and low activity from the region of bistabilty which width varies with the temperature. At low hydrogen pressures (below the τ_A_ line in Fig. 2c), the surface is oxygen-covered and catalytically inactive, due to the suppression of hydrogen adsorption by oxygen [29]. At increasing hydrogen pressure, adsorption of hydrogen and reaction with O depletes the oxygen adlayer and the surface switches to the high activity state (along the τ_A_ line in Fig. 2c). Upon reducing the hydrogen pressure, the system switches to the low activity state (along the τ_B_ line in Fig. 2c). In between τ_A_ and τ_B_, the region of bistability is marked, in which the system is either active or inactive, depending on from which steady state the H_2_ pressure variation originates.

**Fig. 3:**
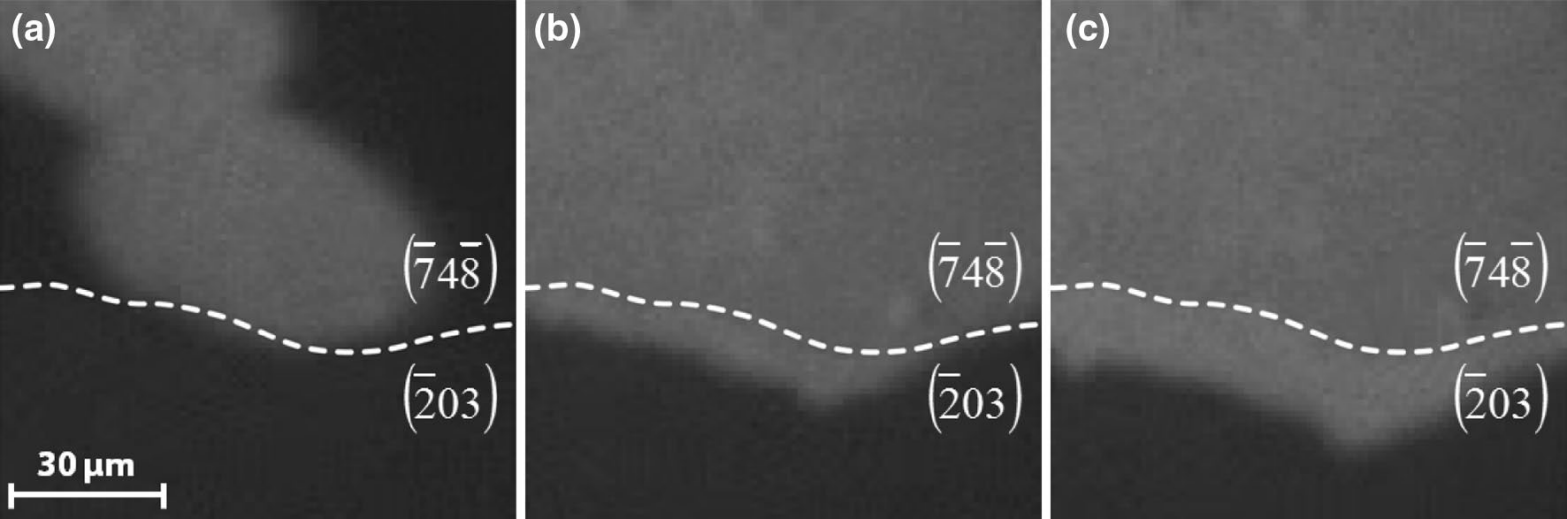
Propagation of a reaction front in H_2_ oxidation across the grain boundary of a polycrystalline Rh foil: **a** a video-frame illustrating the arrival of the hydrogen front at the grain boundary between the $(\bar{7}4\bar{8})$ and $(\bar{2}03)$ domain, marked by the dashed white line; **b** the same but 0.75 s later; **c** the same but 3 s later

The most interesting observation during monitoring the kinetic transitions in H_2_ oxidation was that the reaction fronts unimpededly crossed the grain boundaries (at least under the applied conditions). This is a striking difference to CO oxidation (under comparable conditions), for which the reaction fronts are confined within the individual domains of the polycrystalline sample, as observed for Pt, Pd [8–10] and for the present Rh sample [30]. Figure 3 illustrates this observation: the hydrogen front (bright contrast) spreads from the Rh$(\bar{7}4\bar{8})$ domain towards the Rh$(\bar{2}03)$ domain proceeding smoothly across the grain boundary.

Since the activation energies for CO and H_2_ oxidation on Rh are comparable [31, 32], the reason for the different behaviour of the fronts may originate from the different diffusion properties of carbon monoxide and hydrogen on Rh. In the temperature range of the present study and at comparable submonolayer coverages, the diffusivity of hydrogen on Rh(111) is at least two orders of magnitude higher than that of CO [33]. Accordingly, hydrogen can thus more easily pass through the sub-micrometer “cracks” forming grain boundaries between the domains. Our recent study of CO oxidation on Ar^+^ sputtered Pd surfaces supports this suggestion: on the annealed Pd surface the fronts were confined within the domains, whereas on the same, but additionally Ar^+^ sputtered surface, when the boundary cracks were partially filled with sputtered Pd, the reaction fronts were able to cross the grain boundaries [34].

It is important to note that the propagation of the reaction fronts across the grain boundaries in H_2_ oxidation couples the kinetic transitions on the different domains on the polycrystalline sample. Therefore, the kinetic phase diagram in Fig. 3c represents the whole Rh sample surface, in contrast to CO oxidation, for which such diagrams could be determined for each individual (uncoupled) domain [8, 30].

**Fig. 4:**
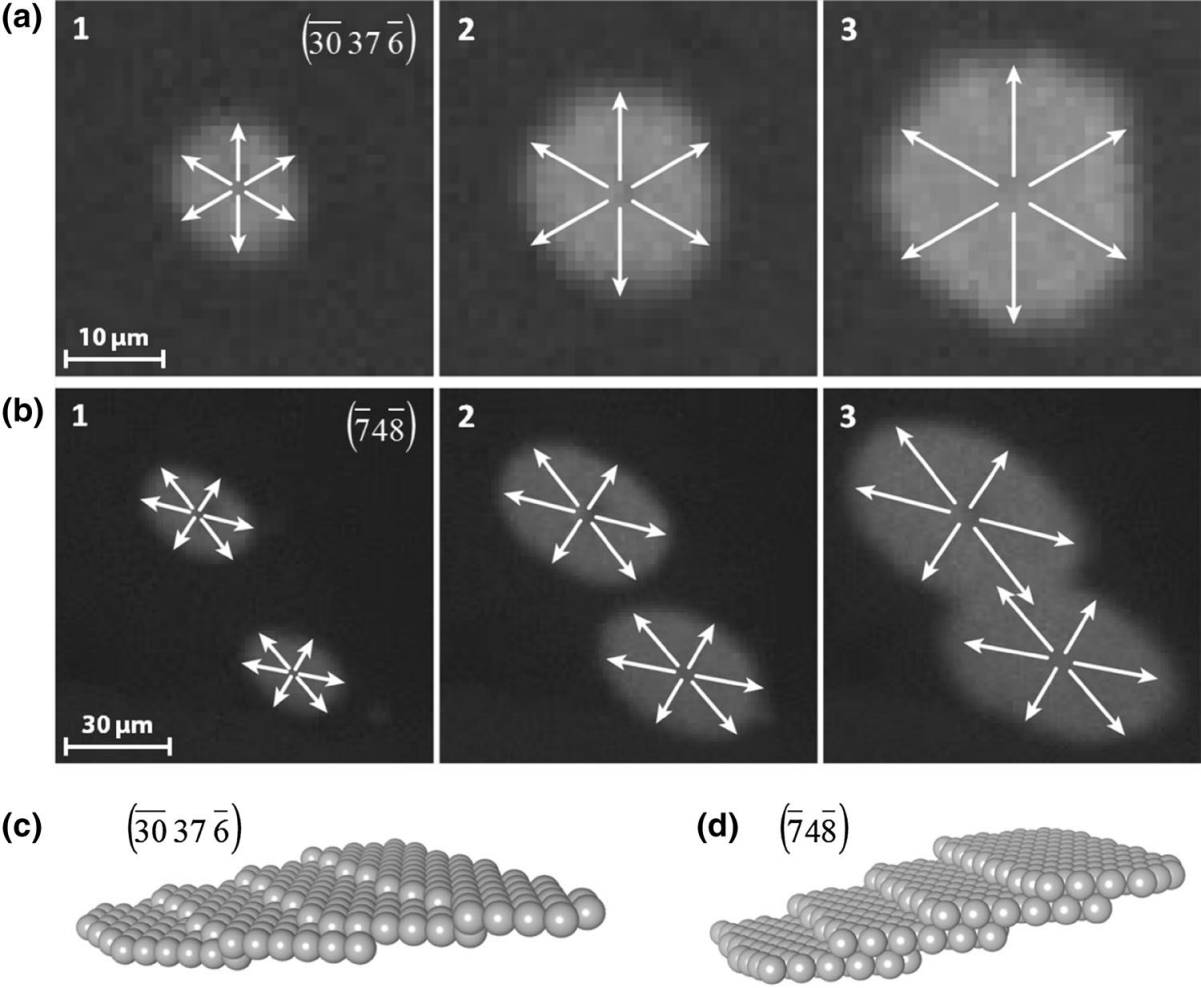
Different modes of front propagation in H_2_ oxidation on stepped Rh surfaces: **a** a circular hydrogen front on the $(\bar{3}\bar{0}37\bar{6})$ surface region of the polycrystalline Rh foil at 473 K, an oxygen pressure of 7.7 × 10^−7^ mbar and a hydrogen pressure of 2.3 × 10^−6^ mbar. The time-interval between the frames is 0.75 s; **b** the same as (a) but on the Rh$(\bar{7}4\bar{8})$ domain, where an elliptic front propagates; **c** ball model of the Rh$(\bar{3}\bar{0}37\bar{6})$ surface; **d** ball model of the Rh$(\bar{7}4\bar{8})$ surface

On the differently stepped Rh surfaces the propagating reaction fronts exhibited different shapes: an approximately circular front was observed on Rh$(\bar{3}\bar{0}37\bar{6})$ (Fig. 4a), whereas on Rh$(\bar{7}4\bar{8})$ an elliptic front was observed (Fig. 4b). This is striking at first, since the $(\bar{3}\bar{0}37\bar{6})$ surface exhibits furrowed (110) terraces, for which one would rather expect an anisotropic front propagation, whereas the $(\bar{7}4\bar{8})$ surface consists of isotropic (111) terraces (Fig. 4d) for which rather a circular front can be expected.

**Fig. 5:**
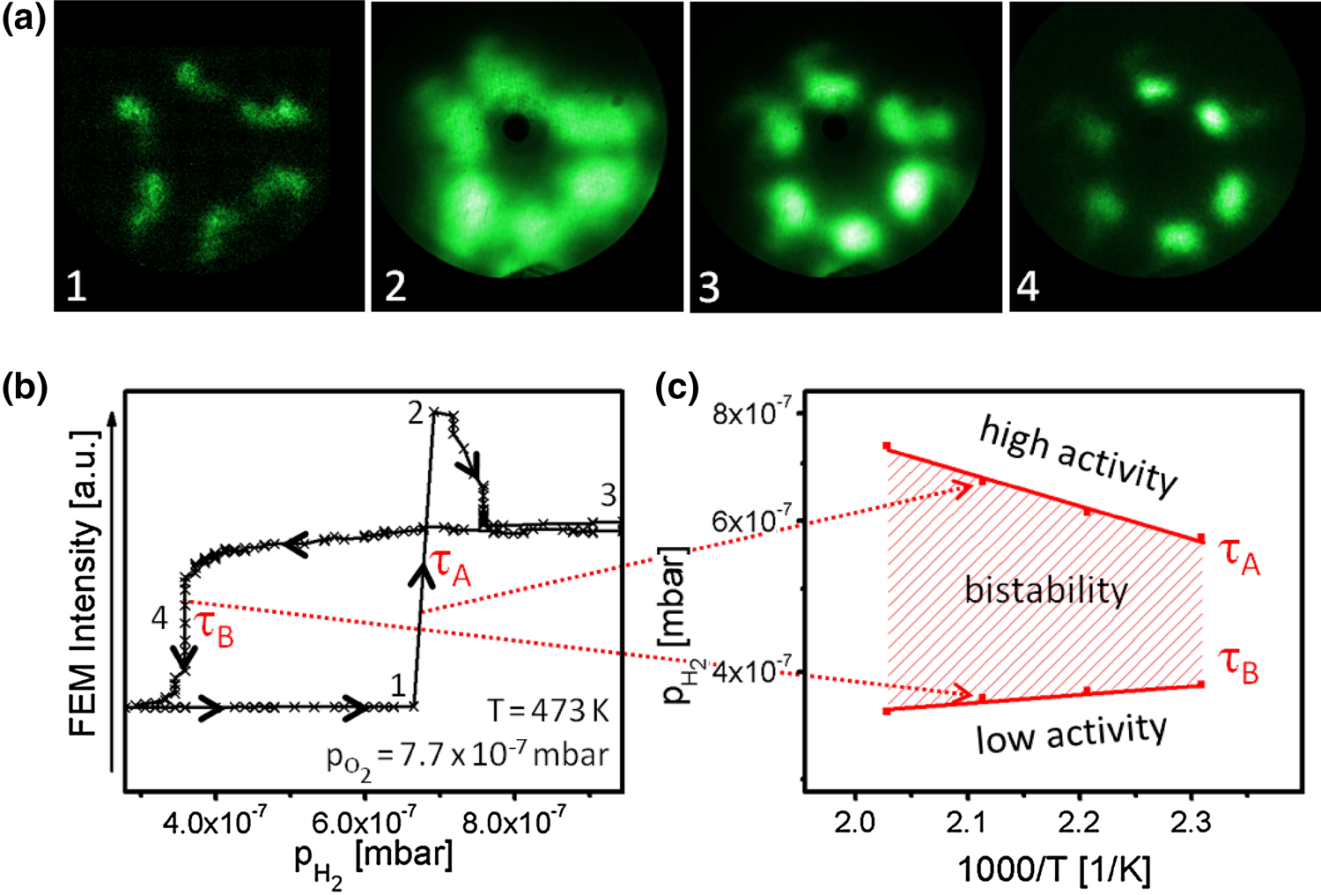
Hydrogen oxidation on a Rh nanotip: **a** examples of FEM video frames taken during the cyclewise variation of the hydrogen pressure at constant oxygen pressure of 7.7 × 10^−7^ mbar and at constant temperature of 453 K; **b** corresponding hysteresis-like curve of the FEM image intensity; τ_A_ and τ_B_ mark the kinetic transitions between the steady states of low and high catalytic activity, the numbers on the curve correspond to the frames in (**a**); **c** τ_A_ and τ_B_ values for different temperatures in the range of 433–493 K and constant oxygen pressure of 7.7 × 10^−7^ mbar, summarised as a kinetic phase diagram

To compare the µm- and nm-scale behaviour of H_2_ oxidation on stepped Rh surfaces, the reaction was also studied by FEM on a Rh nanotip, characterized previously at atomic resolution by FIM (Fig. 1c, d). The experiments were performed in the same manner and under the same conditions as used for the polycrystalline Rh foil. Figure 5 shows the FEM video frames illustrating the kinetic transitions τ_A_ and τ_B_ during the cyclewise variation of the H_2_ pressure at constant O_2_ pressure (7.7 × 10^−7^ mbar) and temperature (453 K), as well as the corresponding hysteresis loop (Fig. 5b) and the resulting kinetic phase diagram. Again, like in the PEEM, the bright contrast corresponds to the high activity state and the dark contrast to the low activity state.

Due to the high hydrogen diffusivity, the reaction is well coupled over the nm-sized apex of the Rh-tip, and the kinetic transitions occur simultaneously (within the camera time-resolution of 0.02 s) on all facets of the apex, i.e. the hysteresis loop and the phase diagram in Fig. 5 represent the whole tip-apex surface.

**Fig. 6:**
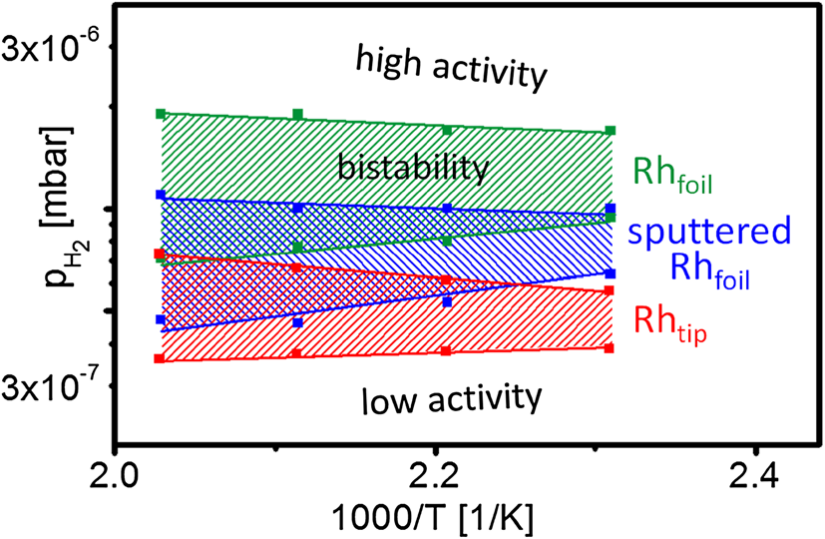
Comparison of the kinetic phase diagrams for hydrogen oxidation on annealed Rh foil (*green*), on the same foil, but additionally Ar^+^ sputtered (*blue*) and on a Rh nanotip (*red*). All diagrams were constructed for the temperature region of 433–493 K and constant oxygen pressure of 7.7 × 10^−7^ mbar

For ease of comparison, the kinetic phase diagrams of the polycrystalline Rh foil and of the Rh nanotip are displayed in Fig. 6, revealing the differences in catalytic behaviour of the µm- and nm-sized systems. As compared to the Rh-tip, the diagram of the Rh foil is shifted to higher (by factor of ~3) hydrogen pressure, indicating the significantly lower tolerance to the poisoning effect of oxygen. Since the step density is known to influence the catalytic behaviour, a Rh sample with a step density which is closer to that of the tip surface was prepared by Ar^+^ sputtering of the present Rh foil (cf. our recent STM observation of the Ar^+^ created steps and defects on Pd [34]). The corresponding phase diagram for such an artificially defected Rh surface is also included in Fig. 6, and is located between those of the stepped Rh foil and of the Rh nanotip. The density of steps and defects on the sputtered Rh surface cannot be exactly determined, but the marked difference from the diagram of the nm-sized Rh tip indicates a significant size effect on the kinetic transitions in H_2_ oxidation. Such an effect, manifested by size-dependent reaction-induced fluctuations, was already observed for CO oxidation on Pt [35, 36], but not yet for H_2_ oxidation.

The higher tolerance of the sputtered Rh foil to the oxygen caused poisoning may result from the increased sticking coefficient of hydrogen in the presence of steps and defects [37]. However, the quantitative estimation of this effect is hardly possible these days due to the lack of the experimental data for the adsorption kinetics of hydrogen on the oxygen precovered Rh surface in the “smooth” and “defected” state.

Regarding the FEM results, a discussion about the potential effect of the electrostatic field (that is required for the field emission imaging) is worthwhile. At first glance, the FEM imaging conditions, where a field of ca. 5 V/nm is usually applied, are not very different from those of FIM, where a field of about 10 V/nm is used to image the surface with O_2_^+^ ions [38].

In fact, a principal difference originates from the field direction: in FIM the tip is positively charged, so that the spatial electron density distribution near the tip surface might be field-modified: the field-free spatial electron density distribution is shifted towards the bulk, depleting the near-surface region [39, 40]. Such a field-modified electron density influences, of course, the interaction of adsorbed molecules with the catalyst surface, as was demonstrated by the atomic scale measurements of the field-dependent binding energy of CO and O_2_ on Pt [41]. Field-modified binding energies of adsorbates influence the adsorption kinetics, shifting the adsorption equilibrium of coadsorbed species. In the case of the catalytic reaction this results in a significant field-induced shift of the kinetic phase diagram as was directly demonstrated for the CO oxidation [42] and for the H_2_ oxidation [43, 44] by in situ FIM of corresponding reactions. Moreover, in the case of positively charged tip, high electric field may even lead to the field-induced reaction-rate oscillations [45]. Therefore, an evaluation of the structure and size effects via a comparison of the FIM results with the data obtained by field-free methods becomes hardly possible.

However, in the case of the negatively charged tip surface used in FEM, the onset of field emission of electrons is achieved at field strengths far below values at which modifications of the electron density can occur. Therefore, the present FEM studies, for which field values of less than 5 V/nm were used, can be considered as quasi field-free, as proven in our previous study of the CO oxidation on Pt using a pulsed high-voltage supply with varying duty pulses [42]. Thus the present FEM results allow, in contrary to the FIM measurements, a direct comparison with the PEEM data without any field-effect corrections.

## Summary

Kinetic measurements of H_2_ oxidation on stepped Rh surfaces were performed using an experimental approach based on the analysis of microscopic (PEEM or FEM) images, obtained in situ during the ongoing reaction. As catalytic model systems, µm-sized high-index domains of a polycrystalline Rh foil and the nm-sized facets of the apex of a Rh nanotip, were used. The reaction was monitored in the 10^−6^ mbar pressure range by PEEM for the crystalline grains of the Rh foil and by FEM for the Rh tip apex, with the latter characterized at atomic resolution by FIM.

A significant difference in the degree of the spatial coupling of H_2_ oxidation on Rh as compared to CO oxidation was detected: in contrast to CO oxidation, for which the reaction is confined within the individual domains, the H_2_ oxidation occurs as spatially coupled for the whole heterogeneous Rh sample (under similar conditions). This is explained by the differing surface diffusion behaviour of hydrogen and CO on Rh. Peculiarities of the hydrogen surface diffusion on stepped Rh surfaces also contribute to the anisotropic reaction front propagation during the kinetic transitions between the steady states of low and high activity.

Kinetic phase diagrams of H_2_ oxidation in the 10^−6^ mbar pressure range were constructed for µm-sized stepped Rh surfaces, for the same surfaces but artificially defected by Ar^+^ sputtering, and for the stepped surfaces of a Rh nanotip. The artificially defected Rh surface appeared to be much more tolerant to the oxygen poisoning of the surface. The nm-sized stepped facets of a Rh nanotip were found, however, even more oxygen tolerant, indicating the existence of a size-effect. Reaction-induced fluctuations might contribute to the observed size-dependent differences in the catalytic behaviour of µm- and nm-sized stepped Rh surfaces.
